# Detection and Impact of *Staphylococcus aureus* Small Colony Variants in Chronic Wounds: A Pilot Study

**DOI:** 10.3390/pathogens14101023

**Published:** 2025-10-09

**Authors:** Eleanna Carris, Klara C. Keim, Landrye Reynolds-Reber, Isaiah K. George, Nicholas Sanford, Rocio Navarro-Garcia, Taylor D. Lenzmeier, Allie Clinton Smith

**Affiliations:** 1 Honors College, Texas Tech University, Lubbock, TX 79410, USA; 2 MicroGenDX, Lubbock, TX 79413, USA; nicholas.sanford@microgendx.com; 3 RTLGenomics, Lubbock, TX 79413, USA; rocio.navarro@rtlgenomics.com; 4 Clinical Microbiology Department, Covenant Medical Center, Lubbock, TX 79410, USA

**Keywords:** *Staphylococcus aureus*, *S. aureus* small-colony variant, clinical diagnostics

## Abstract

A unique phenotype of *S. aureus* called *S. aureus* small-colony variants (SA-SCVs) are a consequential contributor to multiple infectious processes. SA-SCVs are distinguishable from wild-type *S. aureus* (WT-SA) by their small size, slowed growth rate, and altered biochemical reactions; these changes make SA-SCV more difficult to detect from clinical specimens using routine diagnostics. While the clinical environment of chronic wound infections has the potential to stimulate the production of SA-SCVs, studies investigating detection of SA-SCVs in chronic wounds have not been previously conducted. Chronic wound specimens found to harbor *S. aureus* via qPCR screening, and screened for recent aminoglycoside treatment and/or co-infected with *Pseudomonas aeruginosa*, were collected from a specialty wound care clinic in April 2019. In-house enrichment methods alongside culture-dependent and independent diagnostics were utilized to recover and identify SA-SCVs from these chronic wounds. Our investigation determined difficulties in recovering and identifying SA-SCVs during routine diagnostic procedures, and the potential clinical impact of wounds harboring SA-SCVs related to antimicrobial susceptibility.

## 1. Introduction

Chronic wound infections have become a growing worldwide problem due to their severity and vast number of complications, often causing poor patient outcomes. Wounds are considered chronic when healing of the skin in a specific region does not occur in a predictable, timely, and orderly way, or when healing has not been achieved within three months [1]. There are many different types of chronic wounds, such as non-healing diabetic foot ulcers, pressure or decubitus ulcers, venous and arterial ulcers, and non-healing surgical-site infections. These chronic wounds are of great concern, as approximately 6.5 million people in the United States alone are affected by them, and it is estimated that more than $25 billion USD is spent each year by the US healthcare system to treat wound-related complications [2]. Additional estimates also reveal the economic burden of biofilms, with a staggering annual expenditure of approximately $5 trillion USD on biofilm management across various industries [3,4]. Chronic diabetic foot ulcers are globally known to be the most significant wound care problem. It is estimated that diabetic foot ulceration has an annual incidence rate between 1.9% and 4% among diabetics, and a lifetime risk of occurrence between 19% and 34% [5]. Chronic diabetic foot ulcers are also of great concern as 84–85% of diabetic patient amputations are preceded by these ulcers [5,6]. These figures are also expected to increase with the rising prevalence of diabetes [7].

Two of the most common polymicrobial chronic wound pathogens are *Staphylococcus aureus* (SA) and *Pseudomonas aeruginosa* (PA) [8]. These microorganisms are frequently found together in chronic wounds and evidence suggests that these coinfections cause worse patient outcomes than mono-infections [9,10]. A retrospective study found that SA and PA were the most common bacterial pair found in chronic wounds, and are found together in 62% of diabetic wounds [11]. Another study conducted in Denmark estimated that 88–100% of chronic wounds infected with PA were also infected with SA, and the most common resident bacterial species was SA [12]. Additional studies found that a large percentage of nosocomial infections have been due to Methicillin-resistant *S. aureus* (MRSA) [13,14]. Such a high prevalence of SA within chronic wound environments and high levels of antibiotic resistance make treatment of wound infections difficult and co-infections pose serious threats to chronic wound care.

A particularly interesting variant of *Staphylococcus aureus*, known as *Staphylococcus aureus* small colony variant (SA-SCV), was first reported in 1976 [15] and has since been recovered in numerous specimen types such as cystic fibrosis (CF), sepsis, osteomyelitis, foreign-body-associated infections, and skin and soft-tissue infections (SSTIs) [16,17,18,19,20]. Currently, there has been only a single study, published in 2000, that identified SA-SCV from a wound specimen [21]. This study describes a case report of a single wound that was found to be harboring SA-SCVs. It documents the patient’s symptoms, explains how SA-SCV was identified, and reports the treatment used to heal the single wound. However, no literature exists that examines the detection methods or consequences of SA-SCV in chronic wounds.

**Figure 1:**
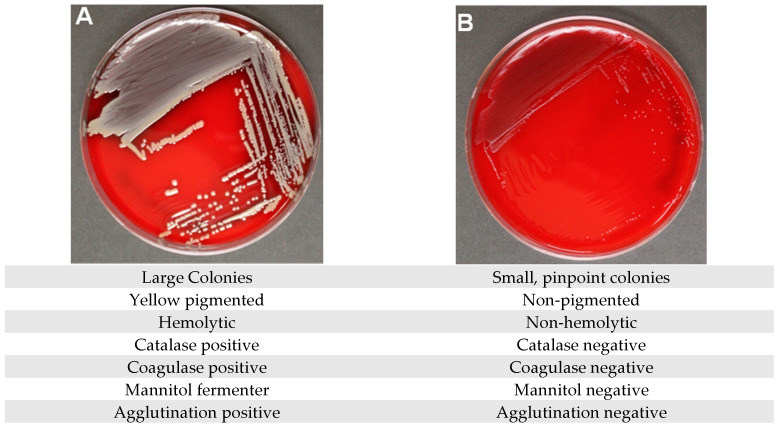
Morphological and biochemical differences exhibited by WT-SA and SA-SCV. Blood Agar Plates after 48 h of incubation at 37 °C, showing a comparison of the colony morphology of WT-SA (**A**) to the SA-SCV phenotype (**B**). Wild-type *S. aureus* with the normal phenotype characterized by yellow pigmentation and hemolysis ((**A**) *S. aureus* Newman parental strain). *S. aureus* small colony variant characterized by pinpoint colonies that are non-pigmented and non-hemolytic ((**B**) *S. aureus* Newman ΔmenB). Images taken by Klara Keim of the TTUHSC Dept. of Surgery and TTU Honors College; strains provided by Catherine Wakeman, TTU Department of Biological Sciences.

SA-SCV is readily distinguishable from the wild-type *Staphylococcus aureus* (WT-SA) due to some defining characteristics. In comparison to WT-SA, the SA-SCV phenotype has much smaller colony sizes that are about one-tenth the size of WT-SA [22] (Figure 1). In addition, SA-SCV has a reduced growth rate, decreased pigmentation, and decreased hemolysis [22]. The slower growth rate of SA-SCVs causes detection of this bacteria to be possible after 48–72 h of incubation, which often leads to overgrowth of WT-SA and other bacterial species that inhibit the detection of SA-SCV [19,21]. SA-SCV’s slower growth rate and differences in biochemical reactions (Figure 1) make it very difficult to identify in a clinical microbiology laboratory, which can lead to misdiagnosis [23]. SA-SCV phenotypes result from mutations that make SA-SCVs unable to synthesize exoproducts such as thymidine, menadione, and/or hemin, which leads to deficiencies in electron transport. Due to the electron-deficient state of SA-SCV, this phenotype is less susceptible to certain antimicrobials, most importantly aminoglycosides, which are commonly used to treat chronic wounds [24]. Exposure to PA exoproducts such as hydrogen cyanide, pyocyanin, and alkyl-hydroxyquinoline N-oxides has been found to stimulate SA-SCV induction [25,26,27,28]. Coinfections of PA and SA-SCV are also more likely to form biofilms, further complicating treatment [29,30].

This study aims to examine the diagnostic procedures used to identify SA-SCVs in patient wound specimens. To achieve this, chronic wound samples were collected from the Southwest Regional Wound Care Center (SWRWCC; Lubbock, TX, USA). Quantitative real-time PCR (qPCR) was used to screen samples for the presence of SA, and samples meeting the inclusion/exclusion criteria were included in this study. We subjected samples to both in-house enrichment protocols specific to SA-SCVs as well as gold-standard culture-dependent diagnostics at Covenant Medical Center Clinical Laboratory Sciences Department (CMC; Lubbock, TX, USA). Lastly, specimens that screened positive for harboring suspected SA-SCVs were sent for confirmatory testing using culture-independent next generation sequencing testing at RTLGenomics (Lubbock, TX, USA). Comparison of the results from different methods of SA-SCV recovery methods and both culture dependent and independent diagnostic procedures could be helpful for improving detection of SA-SCVs in patient specimens and provide insight into the contributing factors involved in SA-SCV infections and their consequences.

## 2. Materials and Methods

### 2.1. Chronic Wound Sampling

Chronic wound specimens as 5 mm punch biopsies from the wound bed post debridement were collected from patients at the Southwest Regional Wound Care Center (SWRWCC; Lubbock, TX, USA) in April 2019 under an IRB-approved protocol #20062347 (approved by Western IRB). In order to be included in the study, participants were screened by having to meet at least two of the following three criteria: (1) previous SA infection (as determined by qPCR), (2) previous SA/PA coinfection (as determined by qPCR), and/or (3) having been prescribed gentamicin (an aminoglycoside) within the last two months. A previous SA/PA coinfection was used as a criterion due to the tendency for SA-SCVs to be stimulated amongst PA infections. A previous gentamicin prescription could also predict the presence of SA-SCV as this bacterial phenotype is stimulated in response to aminoglycoside treatment, rendering the SA-SCV resistant. If a sample was deemed eligible for the study, a section of the tissue specimen was transferred to our laboratory for further testing. In total, 10 specimens meeting the eligibility criteria were obtained and evaluated for this study. Specimens obtained were partitioned into three sections and processed as described below. One portion of tissue samples was frozen in PBS + 50% glycerol and held for future testing if necessary.

### 2.2. Culture-Based Biochemical Diagnostics

One section of whole tissue was transported to Covenant Medical Center Clinical Laboratory Sciences Department (CMC; Lubbock, TX, USA) in thioglycolate broth for a series of culture-based microbiological testing for speciation identification. Wound specimens were processed under standard conditions to obtain a pure culture of suspected pathogen(s), and biochemical testing via the BD Phoenix (Becton Dickinson (BD), Franklin Lakes, NJ, USA) identification panels was performed.

### 2.3. In-House Enrichment

Simultaneously, a second section of whole tissue was processed in-house (TTUHSC Department of Surgery) to enrich specifically for SA-SCVs to enhance recovery. The tissue samples obtained were ground using the Fisherbrand Closed Ultra Tissue Grinder System (Fisher Healthcare, Houston, TX, USA) in thioglycolate broth. The samples were plated in triplicate on multiple agar types to enrich for SA-SCV growth. Determination of suspected SA-SCV colonies on initial in-house screening was based on morphology and growth on the following agars: Blood Agar Plates (BAP, Remel R01200) [31,32], Mannitol Salt Agar (MSA, Remel R01580) [24], BAP +5% Gentamicin (BD BBL L97457) [33], CHROMAgar *S. aureus* (BD BBL 14-432-41) [31], and *S. aureus* ID Agar (SAIDE: bioMerieux 419042) [31]. These agars were selected as the literature describes their ability to detect SA-SCVs, and they are summarized in Supplementary Table S1.

Quadrant streaking was utilized for colony isolation and the plates were incubated at 37 °C, checking at 24, 48, and 72 h post inoculation. After suspected SA-SCVs were identified from the initial screen, the suspected SA-SCV colonies were re-isolated for pure culture on BAP, SAIDE, CHROMAgar, and the original screening plate in triplicate. Once a pure culture of suspected SA-SCV specimen was obtained, the samples were split into three sections once again for extended testing of suspected SA-SCVs as described below.

### 2.4. Testing of Suspected SA-SCVs

Following in-house enrichment, suspected SA-SCV pure culture samples on agar plates were transported to the CMC Clinical Laboratory (Lubbock, TX, USA) for culture-based microbiological identification for speciation by either MALDI-TOF testing or BD Phoenix Gram-positive identification panels (PID) using the BD Phoenix M50 Automated Microbiology System (Becton Dickinson (BD), Franklin Lakes, NJ, USA).

Simultaneously, pure culture samples were also subjected to in-house (TTU Department of Surgery) biochemical reaction determination. Pigmentation and hemolysis were determined by plating on Blood Agar Plates, and mannitol fermentation was determined by plating on Mannitol Salt Agar. Biochemical tests were performed manually for catalase (BD Catalase Reagent Dropper, BD 261203), coagulase (BD BBL™ Rabbit Coagulase Plasma, BD 240827), and agglutination (BD BBL™ Staphyloslide™ Latex Test Kit, BD 240915).

Additionally, suspected SA-SCV pure culture samples on agar plates were also transported to RTLGenomics (Lubbock, TX, USA) for confirmatory testing using next-generation sequencing techniques. DNA extraction was performed using the DNeasy PowerSoil Pro Kit (Qiagen Inc., Valencia, CA, USA) following the manufacturer’s protocol. Where applicable, qPCR for SA-specific genes as well as 16S amplicon sequencing was performed to speciate the suspected SA-SCV samples. For some samples that turned out to be fungal, ITS qPCR was performed for confirmation (fungal identification could have been made from an initial Gram-stain, but specimens did not follow a traditional clinical workflow in this study). Extracted DNA was analyzed on the Roche Light Cycler 480 (LC480; Roche Life Sciences, Basel, Switzerland) for qualitative and quantitative real-time PCR (qPCR) for *Staphylococcus aureus* using Quanta Perfecta Tough Mix (QuantaBio, Beverly, MA, USA) with analyte-specific primers and probes, with LOD being above 30 cycles. The samples were amplified via PCR separately for 16S rRNA regions V1-V3 and V3-V4 using Qiagen HotStarTaq master mix (Qiagen Inc., Valencia, CA, USA) on ABI Veriti thermocyclers (Applied Biosytems, Carlsbad, CA, USA). Amplification products were visualized with eGels (Life Technologies, Grand Island, NY, USA), pooled equimolarly, and size-selected using SPRIselect Reagent (BeckmanCoulter, Indianapolis, IN, USA). Pools were then loaded on an Illumina MiSeq (Illumina, Inc., San Diego, CA, USA) at 10 pM using a MiSeq Reagent kit v3 (600-cycle). Confirmation of the fungal origin of some specimens was achieved by amplification of the ITS1F region via PCR with similar conditions as those described for the 16S rRNA assays, with the corresponding fungal primers.

Pure cultures were also frozen in PBS + 50% glycerol and held once again for further testing if necessary.

**Figure 2:**
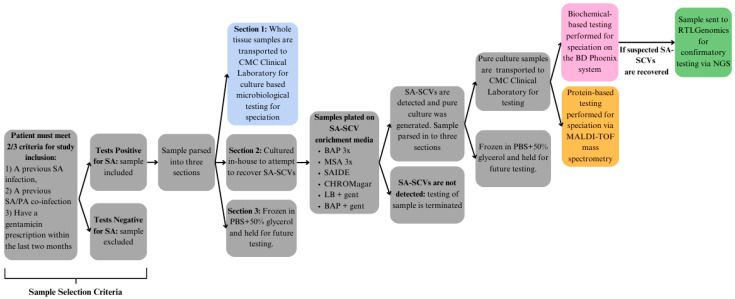
Workflow for SA-SCV identification. Workflow of process to select, enrich, and screen specimens for SA-SCVs through a variety of procedures and tests. Samples were selected based on sample selection criteria and proceeded to move through workflow as specimens continued to meet suspected SA-SCV requirements.

The workflow of this project can be found in Figure 2.

### 2.5. Minimum Inhibitory Concentration (MIC) Testing

Pure cultures of a confirmed SA-SCV, and WT-SA (ATCC 25923) were sent to the CMC Clinical Laboratory for additional MIC testing on the BD Phoenix system. For susceptibility, the BD Phoenix PMIC-108 panel was analyzed by the BD Phoenix M50 Automated Microbiology System. The PMIC-108 contains antibiotics for Gram-positive organisms which includes Ampicillin, Cefazolin, Cefoxitin, Clindamycin, Daptomycin, Erythromycin, Gentamicin, Levofloxacin, Linezolid, Minocycline, Moxifloxacin, Nitrofurantoin, Oxacillin, Penicillin, Quinupristin/Dalfopristin, Rifampin, Tetracycline, Trimethoprim/Sulfamthoxazole, and Vancomycin.

## 3. Results

### Efficacy of Gold-Standard Testing at Detecting SA-SCVs

Three different strains of WT-SA and three strains of known SA-SCVs were tested as positive and negative controls to determine the accuracy of both presumptive identification (PID) on the BD Phoenix system and MALDI-TOF testing at detecting SA-SCVs. While PID testing is based on biochemical results, MALDI-TOF testing is a protein-based analysis which identifies organisms based on protein expression profiles [34].

Following the inclusion criteria, ten chronic wound specimens were selected and evaluated. Initial testing was performed at the Covenant Medical Center (CMC) clinical laboratory (Lubbock, TX, USA), where whole tissue was submitted to the laboratory for analysis based on PID biochemical testing. Of these specimens, it was found that only 4 out of 10 (40%) were found to be harboring SA (Table 1).

**Figure 3:**
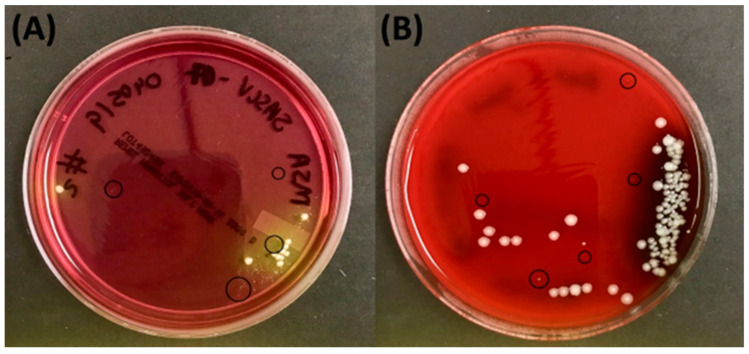
Suspected SA-SCVs from clinical chronic wound specimens on (**A**) MSA and (**B**) BAP+ gentamicin. Images of suspected SA-SCV colonies on agars that select for (**A**) Staphylococci (MSA plate) or (**B**) gentamicin-resistant organisms (BAP+ gentamicin), incubated at 37 °C for 48 h. Suspected SA-SCVs are circled. Example of how suspected SA-SCVs were grown direct from sample alongside colonies that are not of interest in this study, then subsequently isolated for pure culture, and included in downstream analysis.

Whole-tissue samples were also screened in-house in an attempt to recover SA-SCVs by using a variety of enrichment media as described in the Methods section. Most specimens were initially mixed with WT-SA and other wound microorganisms, as shown in Figure 3, and multiple rounds of recovery was necessary to generate pure cultures of suspected SA-SCVs; a colony was treated as a suspected SA-SCV due to colony appearance as well as slow growth rate and appearance on the selected enrichment media as described in Supplementary Table S1. Of the 10 chronic wound samples screened in-house for presence of SA-SCVs, 7 generated a pure culture of a colony suspected to be SA-SCVs. These pure cultures of suspected SA-SCVs were then submitted to CMC for culture-based PID; results are reported in Table 1 (second column), and in-house manual biochemical testing was performed (Supplementary Table S3).

None of the pure cultures of suspected SA-SCVs resulted in the detection of *S. aureus* via culture-based PID on the BD Pheonix system (performed at CMC, Lubbock, TX, USA). Other species of microorganisms such as *Staphylococcus warneri*, *Corynebacterium amycolat/strait*, *Micrococcus luteus*, and *Corynebacterium jeikeium* were detected via this method (Table 1, second column).

Suspected SCV pure-culture samples were also sent for protein-based MALDI-TOF testing (Table 1, third column). For five of the seven specimens tested with both biochemical ID and MALDI-TOF, the results were congruent. For one specimen (specimen 2), biochemical ID identified the culture as *Micrococcus luteus*, while MALDI-TOF identified *Janibacter* spp. In contrast to the pure-culture biochemical tests, this protein-based analysis successfully identified a single *S. aureus* identification (specimen 8), while biochemical identification determined the pure culture to be *S. warneri*.

To confirm the bacterial species of our suspected SA-SCVs, confirmatory testing was performed through RTLGenomics (Lubbock, TX, USA) using next-generation sequencing (NGS) techniques. Reads from these assays ranged from ~90,000–168,000 with a mean length of ~500 bp. First, suspected SCV pure-culture samples were subjected to qPCR testing specific to *S. aureus*. Specimens 2, 7, and 10 were found to not be SA via this testing method, in line with the results from biochemical and MALDI-TOF testing performed at CMC (no further testing of these specimens was performed). Specimens 3 and 9 were subjected to amplicon testing for the ITS gene to detect fungi; amplicons for the ITS gene were detected from these specimens, confirming that these cultures were fungal (although fungal speciation was not performed); this is in line with the biochemical and MALDI-TOF testing performed at CMC. Specimens 2 and 5 were subjected to 16S amplicon sequencing for bacterial speciation and were determined to be *Janibacter* spp. and *Enterococcus faecalis*, respectively; this is in line with the diagnostics performed at CMC (although there were incongruencies between biochemical and MALDI-TOF results for Specimen 2).

**Figure 4:**
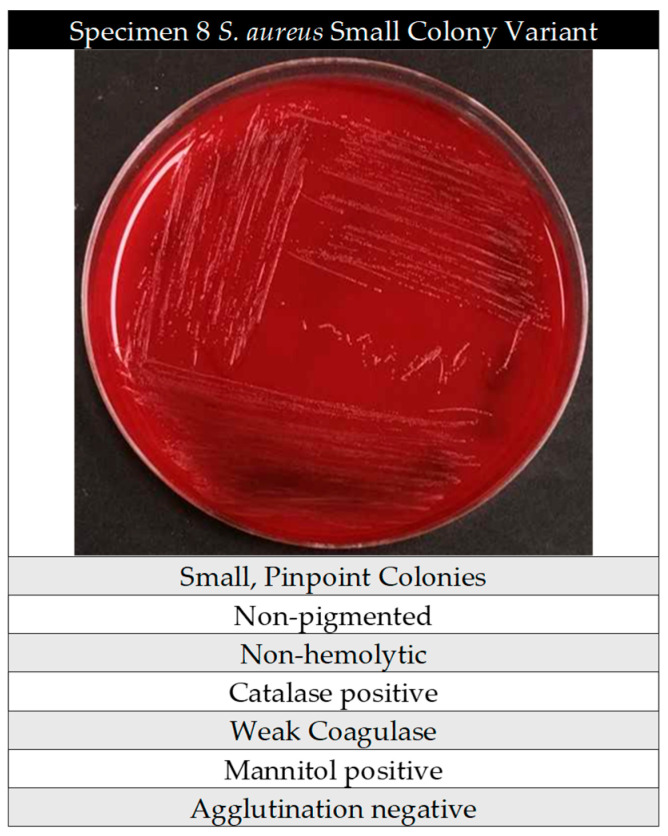
Pure culture of SA-SCV from Specimen 8 and biochemical tests. Image of SA-SCV from Specimen 8, initially recovered on BAP + 5% gentamicin; pure culture generated on BAP. In-house biochemical testing results of SA-SCVs from Specimen 8. Photo courtesy of Klara C. Keim.

Specimen 8 was confirmed to be SA via qPCR for *S. aureus*, and given its altered biochemical reactions and colony appearance, we determine that specimen 8 is in fact an SA-SCV (Figure 4). These data (Table 1) confirmed that SA-SCVs were present in Specimen 8 and found that ~10% (1/10) of our sampling pool contained SA-SCVs.

A pure culture of Specimen 8, the confirmed SA-SCV, was then subjected to MIC testing alongside WT-SA (ATCC 25923) on the BD Phoenix system. Although the MIC machine reported results as ‘No Interpretation’ or NI, for the aminoglycoside amikacin, WT-SA resulted in an MIC value of 2.0 µg/mL, while the Specimen 8 SA-SCV resulted in an MIC value of <256 µg/mL (Supplementary Table S4).

## 4. Discussion

After subjecting known SA-SCVs and tissue samples suspected to be harboring SA-SCVs to a variety of tests, many conclusions about the detection of SA-SCVs can be made.

The PID results from these tests in Supplementary Table S2 support the literature claims about the difficulties of identifying SA-SCVs due to their uncharacteristic biochemical results, with only 1/3 of our known SA-SCVs being identified as *S. aureus* [18,23,35]. MALDI-TOF on the other hand proved much more effective and identified every known SA-SCV as *S. aureus* using protein expression profiles. The differences in the initial PID biochemical testing of known WT-SA and SA-SCV strains (Supplementary Table S2) support the idea that, due to abnormal biochemical reaction signatures, SA-SCVs are very difficult to identify using biochemical reactions. MALDI-TOF, on the other hand, provided a better outcome and successfully identified each strain of known SA-SCVs as *S. aureus* based on protein expression profiles (Supplementary Table S2). These results confirm that biochemical-based diagnostics for SA-SCVs can be lacking, and the authors recommend MALDI-TOF testing for the detection of SA-SCVs in clinical specimens.

Ten chronic wound specimens meeting our inclusion criteria were evaluated. The first testing procedure was submission of a whole tissue sample to the clinical microbiology laboratory at CMC (Lubbock, TX, USA) for routine culture-based PID diagnostics on the BD Pheonix automated system. Of these 10 specimens submitted to the laboratory, only 4 were found to harbor *S. aureus* (Table 1). Given that all specimens initially screened positive for SA through qPCR testing in order to be included in this study, it is suspected that the samples may harbor SA-SCV, but due to their unusual morphology and biochemical reactions, they were not diagnosed as WT-SA using gold-standard culture-dependent methods. Alternatively, it could be that the qPCR methodology used for inclusion criteria screening does detect SA from the specimens, but when tissue is subjected to culture-dependent diagnostics, a low concentration of SA and/or SA-SCVs present in the sample is overgrown by other pathogens and not recovered. In fact, in some of the samples that did not screen positive for SA, coagulase-negative Staphylococcus (CNS) and/or diphtheroids were detected; the literature suggests that SA-SCVs can be misidentified as these organisms due to the biochemical profile of SA-SCV more closely resembling those species instead of WT-SA [36,37].

Pure cultures of suspected SA-SCVs samples following extensive in-house enrichment were tested based on PID biochemical reactions on the BD Phoenix automated system. Following in-house enrichment, we evaluated pure cultures of suspected SCVs from 7 of the 10 chronic wound specimens based on colony appearance and growth rate as described in the Methods section. From PID biochemical testing on the BD Phoenix, none of the pure cultures were determined to be *S. aureus*. Two were found to be fungal, two were determined to be *Corynebacterium* spp., and the others were determined to be *Micrococcus luteus*, *Enterococcus faecalis*, and *Staphylococcus warneri*. In these specimens, the in-house enrichment on specialty agars led to the growth of non *S. aureus* from the original mixed chronic wound specimen; this explains why, for example, Specimen 3 was determined to contain coagulase-negative Staphylococci from the whole tissue submitted to CMC for PID, but following in-house enrichment of the tissue, we submitted a pure culture to CMC for PID which was determined to be *Candida parapsilosis* (Table 1). The original whole tissue likely contained coagulase-negative Staphylococci mixed with the fungi (and the fungi were not reported), but the *Candida parapsilosis* was enriched via our in-house culture methods on specialty agar and identified via PID when submitted as a pure culture.

PID on the BD Phoenix system was used to evaluate automated methods of detection, and misidentified what was later confirmed to be SA-SCV in SCV-08 as *Staphylococcus warneri*. It is possible that, due to WT-SA and SA-SCVs differences in biochemical reactions, automated systems have not traditionally been utilized to identify SA-SCVs and therefore the machine was unable to detect SA-SCV. Specimen 8, originally identified as *Staphylococcus warneri*, showed to be Methicillin-resistant staphylococcus and in-house biochemical testing found that Specimen 8 was non-hemolytic, mannitol-positive, catalase-positive, weak for coagulase, and latex agglutination-negative (Figure 4). When compared to the biochemical characteristics of SA-SCVs in Figure 1, these results differ from the expected behavior of SA-SCVs as they should be negative for all of the previously mentioned tests. Mannitol-positive and catalase-positive differed from the known biochemical characteristics of SA-SCV and may have contributed to their misidentification and incongruent results from each test in the workflow. It is of note that the specimen could have contained both WT-MRSA and SA-SCVs, and the traditional diagnostics only identified the MRSA due to its relative ease of identification. When the whole tissue was originally submitted to CMC for PID, Specimen 8 was determined to be MRSA. It is possible that the specimen contained both MRSA and SCVs, but because of the reduced growth rate of SCVs that MRSA overgrew the SCVs and the SCVs were misidentified due to altered biochemical reactions.

The fact that MALDI-TOF is based on protein expression profiles instead of biochemical reactions provides more insight into the difficulty of clinical detection of SA-SCVs. MALDI-TOF successfully identified both the known SA-SCV samples (Supplementary Table S2) and the confirmed SA-SCV pure culture of Specimen 8 (Table 1) as *S. aureus*. This is a positive indication that MALDI-TOF has the capabilities to detect SA-SCVs as SA, as it also correctly identified WT-SA as well. However, the distinction between WT-SA and SA-SCVs continues to be problematic as even the MALDI-TOF testing used in this study did not distinguish between WT-SA and SA-SCVs. However, the fact that despite its abnormalities SA-SCVs could be identified as SA through MALDI-TOF provides some evidence that MALDI-TOF could have the capability of diagnosing SA-SCVs in the future, if the machine could be optimized to distinguish between WT-SA and SA-SCVs based on protein expression. Currently, this method encompasses WT-SA and SA-SCVs as *S. aureus*, but in the future this distinction could be added to improve detection of SA-SCVs.

Even though MALDI-TOF testing was able to classify SA-SCVs as SA, this was achieved after extensive in-house enrichment to provide a pure sample of suspected SA-SCVs. This indicates that even if using MALDI-TOF, it would most likely prove difficult to identify SA-SCVs directly from a wound. The necessity of enrichment of SA-SCVs is due to a variety of factors such as the polymicrobial nature of most chronic wounds in which the comparatively small colonies and slow growth rate of SA-SCVs will most likely be outgrown by other microorganisms.

The identification of SCVs in clinical samples, including wounds, proves to be difficult but important. Results from MIC testing of WT-SA and SA-SCV (Specimen 8) indicated SA-SCVs’ significantly reduced susceptibility to the aminoglycoside amikacin, further exemplifying their ability to exhibit antimicrobial recalcitrance to a variety of antibiotics, particularly to aminoglycosides, and their higher tendency to form biofilms compared to WT-SA make the identification of SCVs crucial in clinical settings [22,38,39]. Whether a wound is harboring SCVs with or without WT-SA, missing an SCV diagnosis has the potential to negatively impact patient prognosis and has been linked to chronic and recurrent infections [22,40]. Their presence in wound samples and their detection complications highlight a need for accurate detection methods in clinical settings to properly identify and treat SCV infections [36].

Extensive in-house enrichment was conducted from whole-tissue samples in an attempt to recover SA-SCVs from polymicrobial mixed-patient wound specimens. Due to their reduced growth rate and known difficulty in recovery in a laboratory setting, it is possible that additional wound specimens in our study harbored SCVs but were not recovered by our laboratory methods for a variety of biological and technical reasons. While we feel that our results are an important addition to the broader literature about SA-SCVs in the chronic wound environment, sweeping conclusions about their prevalence and impact are difficult to make from a study with such a small sample size. However, we do feel that our findings warrant additional studies to investigate SA-SCVs in chronic wounds on a larger scale.

The data from the culture-based microbiological testing and the biochemical-based testing of pure cultures of suspected SA-SCVs performed by the BD Phoenix system (Table 1) supports the understanding that SA-SCVs prove problematic to identify using gold-standard diagnostics due to their abnormal characteristics such as a slower growth rate and differences in biochemical reactions compared to WT-SA. While MALDI-TOF was more successful at identifying SA-SCVs as SA, testing relied on enrichment of SA-SCVs from a wound tissue sample and therefore identification without deliberately seeking a SA-SCV identification could prove difficult. Although only using a sample of ten wounds, these data indicate the prevalence of SA-SCVs within chronic wound environments after finding that ~10% of the samples tested positive for harboring SA-SCVs. Additional large-scale studies need to be conducted to determine the true prevalence of SA-SCVs in chronic wounds, as well as further investigation into the negative impact of SA-SCV infection, especially in those that go misdiagnosed, is important in terms of patient prognosis and treatment efficacy.

## Figures and Tables

**Table 1:** Results of series of tests to identify SA-SCVs.

Sample	PID from Whole Tissue Submitted to CMC (Lubbock, TX, USA) Evaluated on BD Phoenix	PID of Pure Cultures Evaluated on BD Phoenix (CMC, Lubbock, TX, USA)	Pure Culture Evaluated on MALDI-TOF Testing (CMC, Lubbock, TX, USA)	Confirmatory Testing Evaluated Using NGS Techniques (RTLGenomics, Lubbock, TX, USA)
Specimen 1	Coagulase-negative Staphylococci, diphtheroids	N/A	N/A	N/A
Specimen 2	Coagulase-negative Staphylococci, MRSA	*Micrococcus luteus*	*Janibacter hoylei*	*Janibacter* spp. (16S amplicon); <LOD 16S qPCR
Specimen 3	Coagulase-negative Staphylococci	*Candida parapsilosis*	*Yeast* spp.	Fungus, unspecified (ITS amplicon)
Specimen 4	MRSA	N/A	N/A	N/A
Specimen 5	MRSA	*Enterococcus faecalis*	*Enterococcus faecalis*	*E. faecalis* (16S amplicon)
Specimen 6	Coagulase-negative Staphylococci	N/A	N/A	N/A
Specimen 7	Coagulase-negative Staphylococci, diphtheroids	*Corynebacterium jeikeium*	*Corynebacterium jeikeium*	<LOD SA qPCR
Specimen 8	MRSA	*Staphylococcus warneri*	*S. aureus*	*S. aureus* SA qPCR
Specimen 9	Gram negative rods (Not *Pseudomonas* spp.)	*Candida parapsilosis*, *Acinetobacter baumannii*	*Candida parapsilosis*	Fungus, unspecified (ITS amplicon)
Specimen 10	Diphtheroids	*Corynebacteria amycolat/ striat*	*Corynebacterium striatum* group	<LOD SA qPCR

Column 1 (blue): Results of whole tissue submitted to the Covenant Medical Center (CMC) clinical laboratory in thioglycolate broth for culture-based microbiological presumptive identification (PID) using the BD Phoenix automated system. Column 2 (pink): pure culture PID identification of suspected SA-SCV following in-house enrichment (TTUHSC) using the BD Phoenix system. Column 3 (orange): where applicable, protein-based MALDI-TOF testing performed on pure cultures of suspected SA-SCV following in-house enrichment (TTUHSC). Column 4 (green): Confirmatory testing using next-generation sequencing at RTLGenomics; where applicable *S. aureus*-specific qPCR testing (SA qPCR), 16S amplicon sequencing, or ITS amplicon sequencing was performed to detect *S. aureus*, and speciate bacteria and fungus, respectively. N/A reported for specimens that were not subjected to specific testing.

## Data Availability

Data are contained within the article.

## Supplementary Materials

The following supporting information can be downloaded at: https://www.mdpi.com/article/10.3390/pathogens14101023/s1, Table S1: Agars used for the in-house enrichment and recovery of SA-SCV; Table S2: Wild Type SA and Known SA-SCV Controls; Table S3: Biochemical Reactions for Suspected SA-SCV colonies from chronic wound specimens; Table S4: Results of Amikacin Minimum Inhibitory Concentration (MIC).

## Abbreviations

The following abbreviations are used in this manuscript:

SA-SCV	*S. aureus* Small-Colony Variant
MALDI-TOF	Matrix-Assisted Laser Desorption/Ionization Time-of-Flight mass spectrometry
