# Supplementary material for: Detection and Impact of Staphylococcus aureus Small Colony Variants in Chronic Wounds: A Pilot Study
Source: Pathogens. 2025 Oct 9;14(10):1023. doi: 10.3390/pathogens14101023 (PMC12567352; doi:10.3390/pathogens14101023)
Supplement: Supplementary file 1 [file pathogens-14-01023-s001.zip › pathogens-3859999-supplementary.pdf]

Supplementary Table S1: Agars used for the in-house enrichment and recovery of SA-SCV. Citations for each agar represent literature evidence and interpretation of SA-SCV on each agar**.**

Agar	Interpretation of SA-SCV
BAP[31, 32]	Small/pinpoint, non-pigmented, non-hemolytic colonies; hemolysis determined after extended incubation
MSA[24]	Small/pinpoint, non-pigmented, and non-mannitol fermenting colonies; mannitol fermentation determined after extended incubation
BAP +5% Gentamicin[33]	Similar growth patterns of SA-SCVs as regular BAPs, but should have been more selective due to SA-SCVs gentamicin tolerance (other gentamicin resistant and/or tolerant colonies could also grow on this agar)
CHROMAgar *S. aureus*[31]	Pinpoint mauve/pink colonies (compared to blue or colorless colonies of non-Staphylococci)
SAIDE[31]	Pinpoint mauve/pink colonies. However, this agar should have been more selective for SA-SCVs compared to the CHROMAgar due to the enrichment of thymidine which was thought to improve recovery of SA-SCVs.

Supplementary Table S2: Wild Type SA and Known SA-SCV Controls. Results of biochemical-based testing performed for speciation on BD Phoenix system and MALDI-TOF testing of three known strains of WT-SA and three known strains of SA-SCVs. WT-SA strains were purchased from American Type Culture Collection (ATCC), and the SA Newman and SA-SCV mutant strains were provided by Catherine Wakeman (Biological Sciences, Texas Tech University, Lubbock, TX).

Controls	PID (biochemical BD Phoenix)	MALDI-TOF (protein-based)
WT-SA ATCC 25923	*S. aureus*	*S. aureus*
WT-SA ATCC 29213	*S. aureus*	*S. aureus*
WT-SA Newman (SASCV mutant parent strain)	*S. aureus*	*S. aureus*
SASCVΔhem	Gram positive cocci (plate mixed), *Aerococcus viridans*	*S. aureus*
SASCVΔcyto	*S. aureus*	*S. aureus*
SASCVΔmen	*S. capitis*	*S. aureus*

Supplementary Table S3: Biochemical Reactions for Suspected SA-SCV colonies from chronic wound specimens

	Biochemical Tests				
Sample Name	Hemolysis	Mannitol	Catalase	Coagulase	Latex Agglutination
WT-SA (expected)*	+	+	+	+	+
Specimen 2	+	+	Weak +	-	-
Specimen 3	Weak +	-	-	-	-
Specimen 5	Weak +	+	-	Weak +	-
Specimen 7	+	+	+	-	-
Specimen 8	-	+	+	Weak +	-
Specimen 9	-	-	-	Weak +	-
Specimen 10	+	-	+	-	-

*Laboratory strain SA31 was utilized to confirm expected biochemical reaction results of WT-SA

Supplementary Table S4: Results of Amikacin Minimum Inhibitory Concentration (MIC)

[redacted]	[redacted]
2µg/ml	>256µg/ml

Results of Covenant Medical Center clinical laboratory minimum inhibitory concentration (MIC), as tested on the BD Pheonix automated system, for susceptibility to amikacin, a commonly used aminoglycoside.
